# Phytonanoparticles as novel drug carriers for enhanced osteogenesis and osseointegration

**DOI:** 10.1186/s11671-024-04164-9

**Published:** 2025-01-16

**Authors:** Nandita Suresh, Nebu George Thomas, Matti Mauramo, Tuomas Waltimo, Timo Sorsa, Sukumaran Anil

**Affiliations:** 1 Department of Oral and Maxillofacial Diseases, Helsinki University and University Hospital, Helsinki, Finland; 2 Pushpagiri Institute of Medical Sciences and Research Centre, Medicity, Perumthuruthy, Tiruvalla, Kerala India; 3 Department of Pathology, University of Helsinki and Helsinki University Hospital, Helsinki, Finland; 4 Faculty of Medicine, University of Basel, Basel, Switzerland; 5 Department of Oral Diseases, Karolinska Institutet, Huddinge, Sweden; 6 Oral Health Institute, Hamad Medical Corporation, Doha, Qatar; 7 College of Dental Medicine, Qatar University, Doha, Qatar

**Keywords:** Phytonanoparticles, Bone regeneration, Osseointegration, Nanotechnology, Tissue engineering, Drug delivery, Gold nanoparticles, Silver nanoparticles, Zinc oxide nanoparticles

## Abstract

Phytonanoparticles have emerged as a promising class of biomaterials for enhancing bone regeneration and osseointegration, offering unique advantages in biocompatibility, multifunctionality, and sustainability. This comprehensive review explores the synthesis, characterization, and applications of phytonanoparticles in bone tissue engineering. The green synthesis approach, utilizing plant extracts as reducing and stabilizing agents, yields nanoparticles with intrinsic bioactive properties that can synergistically promote osteogenesis. We examine the mechanisms by which phytonanoparticles, particularly those derived from gold, silver, and zinc oxide, influence key molecular pathways in osteogenesis, including RUNX2 and Osterix signaling. The review discusses advanced strategies in phyto-nanoparticle design, such as surface functionalization and stimuli-responsive release mechanisms, which enhance their efficacy in bone regeneration applications. Preclinical studies demonstrating improved osteoblast proliferation, differentiation, and mineralization are critically analyzed, along with emerging clinical data. Despite promising results, scalability, standardization, and regulatory approval challenges persist. The review also addresses the economic and environmental implications of phyto-nanoparticle production. Looking ahead, we identify key research directions, including developing personalized therapies, combination approaches with stem cells or gene delivery, and long-term safety assessments. By harnessing the power of plant-derived nanomaterials, phytonanoparticles represent an innovative approach to addressing the complex challenges of bone regeneration, with potential applications spanning dental, orthopedic, and maxillofacial surgery.

**Graphical abstract:**

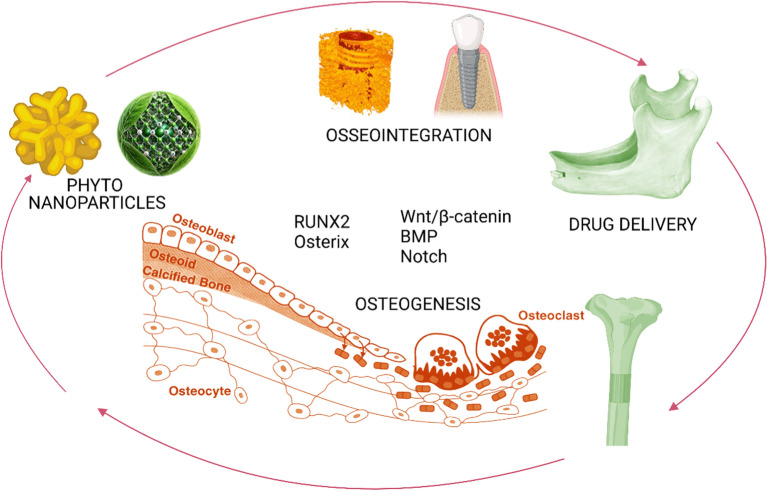

## Introduction

Bone tissue engineering has emerged as a promising field in regenerative medicine, offering potential solutions for repairing and regenerating bone defects caused by trauma, disease, or congenital abnormalities [1]. Despite significant advancements, several challenges persist in achieving optimal bone regeneration and implant integration. These challenges include the need for sustained and targeted delivery of bioactive molecules, promotion of vascularization, and enhancement of osteoblast proliferation and differentiation [2]. One of the primary obstacles in bone tissue engineering is the difficulty in delivering therapeutic agents to the target site with controlled release kinetics. Traditional drug delivery methods often result in rapid clearance or degradation of bioactive molecules, necessitating high doses that can lead to systemic side effects [3]. Additionally, the complex microenvironment of bone tissue, with its dense extracellular matrix and limited blood supply, poses significant barriers to effective drug delivery [4, 5].

Another critical challenge is the promotion of osseointegration, particularly in the context of dental and orthopedic implants. Achieving stable and long-lasting integration between the implant surface and surrounding bone tissue is crucial for the success of these interventions [6]. However, implant surface properties, local inflammation, and impaired osteoblast function can hinder osseointegration, leading to implant failure and needing revision surgeries [7]. In recent years, nanotechnology has offered promising solutions to address these challenges in bone tissue engineering. Nanoparticle-based drug delivery systems have shown potential in providing controlled and sustained release of therapeutic agents and enhancing their stability and bioavailability [8, 9]. Among the various types of nanoparticles, phytonanoparticles have gained significant attention due to their unique properties and potential advantages in biomedical applications.

Phytonanoparticles, also known as green or plant-based nanoparticles, are nanoscale materials synthesized using plant extracts or plant-derived compounds [10]. These nanoparticles offer several potential advantages over conventionally synthesized nanoparticles, including enhanced biocompatibility, reduced toxicity, and bioactive phytochemicals that may confer additional therapeutic benefits [11]. The synthesis of phytonanoparticles typically involves the reduction of metal salts by plant extracts, resulting in the formation of nanoparticles stabilized by plant-derived biomolecules [12]. The potential of phytonanoparticles in drug delivery for bone regeneration lies in their ability to encapsulate and deliver various therapeutic agents, including growth factors, small molecules, and genetic material [13]. Moreover, the surface properties of phytonanoparticles can be tailored to enhance their interaction with bone cells and promote their uptake at the target site. Phytochemicals on the nanoparticle surface may also contribute to their osteogenic and anti-inflammatory properties, potentially enhancing bone formation and reducing adverse reactions [14, 15].

This review article aims to elucidate the current state and potential of phytonanoparticles as innovative drug carriers for enhanced osteogenesis and osseointegration. The review's multifaceted objectives encompass: (1) a thorough examination of the composition, synthesis methods, and characterization techniques of phytonanoparticles pertinent to bone tissue engineering applications; (2) an in-depth exploration of the mechanisms by which phytonanoparticles enhance osteogenesis and osseointegration, with a particular focus on their interactions with bone cells and the bone microenvironment; (3) a critical discussion of recent advancements in the design and fabrication of phyto-nanoparticle-based drug delivery systems for bone regeneration, including cutting-edge surface functionalization strategies and stimuli-responsive release mechanisms; (4) a systematic review of preclinical and clinical studies investigating the efficacy of phyto-nanoparticle-mediated drug delivery in enhancing bone formation and implant integration; and (5) a comprehensive analysis of the challenges and future perspectives in developing and translating phyto-nanoparticle-based therapies for bone tissue engineering, addressing crucial aspects such as scalability, regulatory considerations, and environmental implications. By addressing these interconnected objectives, this review seeks to provide researchers and clinicians with a holistic understanding of the transformative potential of phytonanoparticles in advancing bone tissue engineering while simultaneously identifying key areas for future research and development in this promising and rapidly evolving field.

## Phytonanoparticles: composition, synthesis, and characterization

### Definition and types of phytonanoparticles

Phytonanoparticles, also known as green or plant-based nanoparticles, are nanoscale materials synthesized using plant extracts or plant-derived compounds. These nanoparticles typically range in size from 1 to 100 nm and can be derived from various plant parts, including leaves, roots, stems, fruits, and seeds. The defining characteristic of phytonanoparticles is using plant-derived biomolecules as reducing and stabilizing agents in their synthesis, which can impart unique properties to the resulting nanoparticles [16].

Types of Phytonanoparticles can be classified based on their composition:

1. Metallic phytonanoparticles: These include gold (AuNPs), silver (AgNPs), copper (CuNPs), and other metal nanoparticles synthesized using plant extracts.
2. Metal oxide phytonanoparticles: Examples include zinc oxide (ZnO), iron oxide (Fe3O4), and titanium dioxide (TiO2) nanoparticles.
3. Biogenic silica nanoparticles: Derived from plants rich in silica, such as rice husk and bamboo leaves.
4. Carbon-based phytonanoparticles, including carbon dots and nanotubes, are synthesized using plant extracts.
5. Polymer-based phytonanoparticles: Nanoparticles composed of natural polymers like cellulose, chitosan, or starch, often modified or synthesized using plant-derived compounds.

### Synthesis methods and green chemistry approach

The synthesis of phytonanoparticles adheres to green chemistry principles, emphasizing environmentally friendly processes and renewable resources. This "green synthesis" approach involves reducing metal salts or precursors using plant extracts, both reducing and stabilizing agents. The process typically involves several key steps: First, plant material is processed and extracted to obtain a phytochemical-rich solution [17]. Concurrently, metal salt precursors (e.g., HAuCl4 for gold nanoparticles, AgNO3 for silver nanoparticles) are prepared in suitable solvents. The plant extract is mixed with the precursor solution, usually at room temperature or with mild heating, allowing phytochemicals to reduce metal ions and form nanoparticles. Biomolecules from the plant extract, including proteins, polyphenols, and polysaccharides, coat the nanoparticles, providing stability and preventing aggregation [18]. Finally, the synthesized nanoparticles are purified through centrifugation, filtration, or dialysis. Various factors influence this process, including the plant species and part used, extraction method, concentrations of reactants, pH, temperature, reaction duration, and physical parameters like stirring speed. Recent advancements have enhanced the efficiency and scalability of phyto-nanoparticle synthesis, including microwave-assisted techniques for rapid and uniform production, continuous flow systems for large-scale manufacturing, and photoinduced synthesis utilizing sunlight or artificial light sources. These innovations are expanding the potential applications of phytonanoparticles while maintaining their eco-friendly nature [18].

### Characterization techniques and key physicochemical properties

**Fig. 1:**
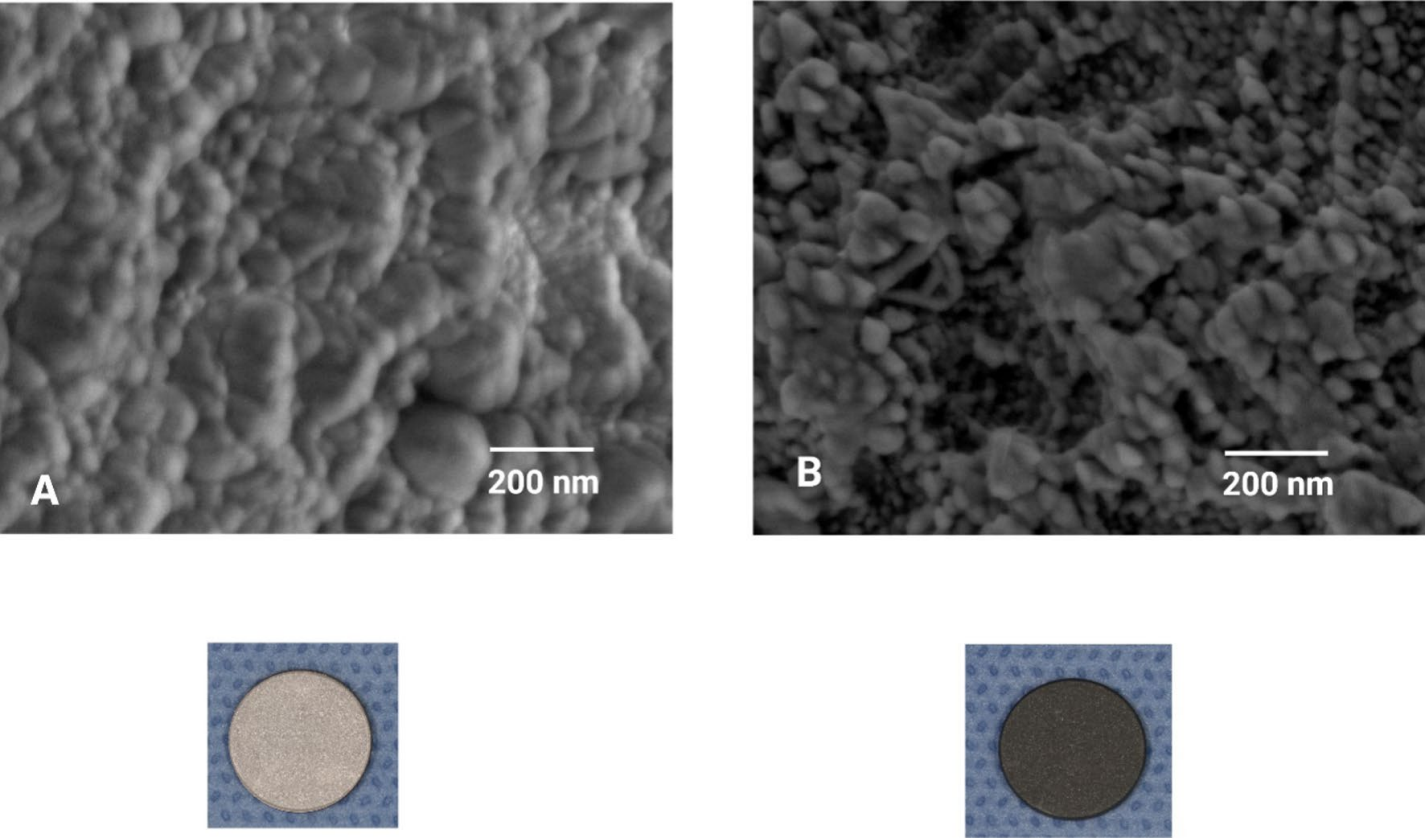
SEM image of the Curcumin-coated titanium disc (A) and HA-coated titanium disc

Characterization of Phytonanoparticles is essential for elucidating their properties and potential applications, employing diverse analytical techniques. UV–visible spectroscopy confirms nanoparticle formation and estimates particle size based on surface plasmon resonance peaks. In contrast, high-resolution morphology and size distribution imaging is achieved through Transmission Electron Microscopy (TEM) [19]. Scanning Electron Microscopy (SEM) provides insights into surface topography and composition (Fig. 1), complemented by Dynamic Light Scattering (DLS) measurements of hydrodynamic size and zeta potential, indicative of solution stability [20]. Crystalline structure and phase composition are determined via X-ray Diffraction (XRD), while Fourier Transform Infrared Spectroscopy (FTIR) identifies functional groups and surface biomolecules. Elemental composition is analyzed using Energy Dispersive X-ray Spectroscopy (EDX), and Thermogravimetric Analysis (TGA) assesses thermal stability and quantifies organic coating. These techniques collectively elucidate key physicochemical properties of Phytonanoparticles, including size and size distribution, shape and morphology, surface charge and zeta potential, crystallinity, surface area and porosity, optical properties (such as surface plasmon resonance), stability in various media, and the phytochemical composition of the surface coating. This comprehensive characterization is crucial for understanding the behavior and potential applications of Phytonanoparticles in diverse biological and environmental contexts [21].

### Comparison of phytonanoparticles with conventional nanoparticles

Phytonanoparticles offer numerous advantages over conventionally synthesized nanoparticles, positioning them as a promising class of nanomaterials for various applications, including drug delivery and tissue engineering. Their eco-friendly synthesis utilizes renewable resources with reduced energy input, while the natural phytochemical surface coating enhances biocompatibility and reduces toxicity compared to chemically synthesized counterparts [22]. This phytochemical coating also imparts multifunctionality, potentially providing additional biological activities such as antioxidant or anti-inflammatory properties and excellent colloidal stability without additional capping agents [23]. Moreover, plant-based synthesis can be more cost-effective than traditional chemical methods, particularly for large-scale production. However, the development of Phytonanoparticles is challenging. Variations in plant composition can lead to batch-to-batch variability in nanoparticle properties, affecting reproducibility. Scaling up synthesis while maintaining consistent quality presents difficulties, and separating nanoparticles from plant debris and unreacted components may require additional processing steps [24]. Despite these challenges, the unique properties derived from their plant-based synthesis, including their green synthesis approach, potential for multifunctionality, and enhanced biocompatibility, make Phytonanoparticles attractive candidates for biomedical applications. Addressing the challenges in reproducibility and scalability will be crucial for their widespread adoption. Still, the potential benefits of these innovative nanomaterials warrant continued research and development efforts in the field [25]. The table concisely compares key characteristics between phytonanoparticles and conventional nanoparticles, highlighting their advantages and challenges (Table 1).

**Table 1:** Comparison of key characteristics of Phytonanoparticles and Conventional Nanoparticles

Comparison of phytonanoparticles and conventional nanoparticles		
Characteristics	Phytonanoparticles	Conventional nanoparticles
Synthesis method	Green synthesis using plant extracts	Chemical or physical methods
Reducing/capping agents	Natural phytochemicals	Synthetic chemicals
Environmental impact	Low, eco-friendly	Potentially higher, may use toxic chemicals
Biocompatibility	Generally higher due to natural coating	Variable; may require additional modification
Multifunctionality	Inherent due to phytochemical coating	Often requires additional functionalization
Cost	Potentially lower, especially at the scale	Variable can be high for some methods
Reproducibility	Can be challenging due to plant variability	Generally, more consistent
Scalability	Promising but can be challenging	Well-established for many types
Purification	May require additional steps	Well-established methods
Characterization	Can be complex due to diverse surface chemistry	Generally, more straightforward
Size control	Moderate, influenced by plant extract	High, precise control in many methods
Shape control	Limited, often spherical	High, various shapes possible
Application range	Broad, with potential for novel applications	Well-established for many applications

## Mechanisms of osteogenesis and osseointegration

### Overview of bone formation and remodeling processes

Bone tissue exhibits a remarkable dynamism, undergoing continuous remodeling throughout an individual's lifetime. This intricate process involves the coordinated actions of three main cell types: osteoblasts (bone-forming cells), osteoclasts (bone-resorbing cells), and osteocytes (mature bone cells embedded within the bone matrix). The bone remodeling cycle progresses through four distinct phases: activation, where osteoclast precursors are recruited to the bone surface and differentiate into mature osteoclasts; resorption, during which osteoclasts create an acidic microenvironment and secrete proteolytic enzymes to dissolve the existing bone matrix; reversal, where mononuclear cells prepare the bone surface for new bone formation; and formation, in which osteoblasts synthesize new bone matrix (osteoid) and regulate its mineralization [26–28]. The bone formation itself occurs through two primary mechanisms: intramembranous ossification, typical of flat bones, involving the direct differentiation of mesenchymal stem cells into osteoblasts, and endochondral ossification, characteristic of long bones, which involves the formation of a cartilage template that is gradually replaced by bone tissue. This complex interplay of cellular activities and ossification processes ensures bone tissue's continuous adaptation and renewal throughout life [29].

### Key molecular pathways in osteogenesis

**Fig. 2:**
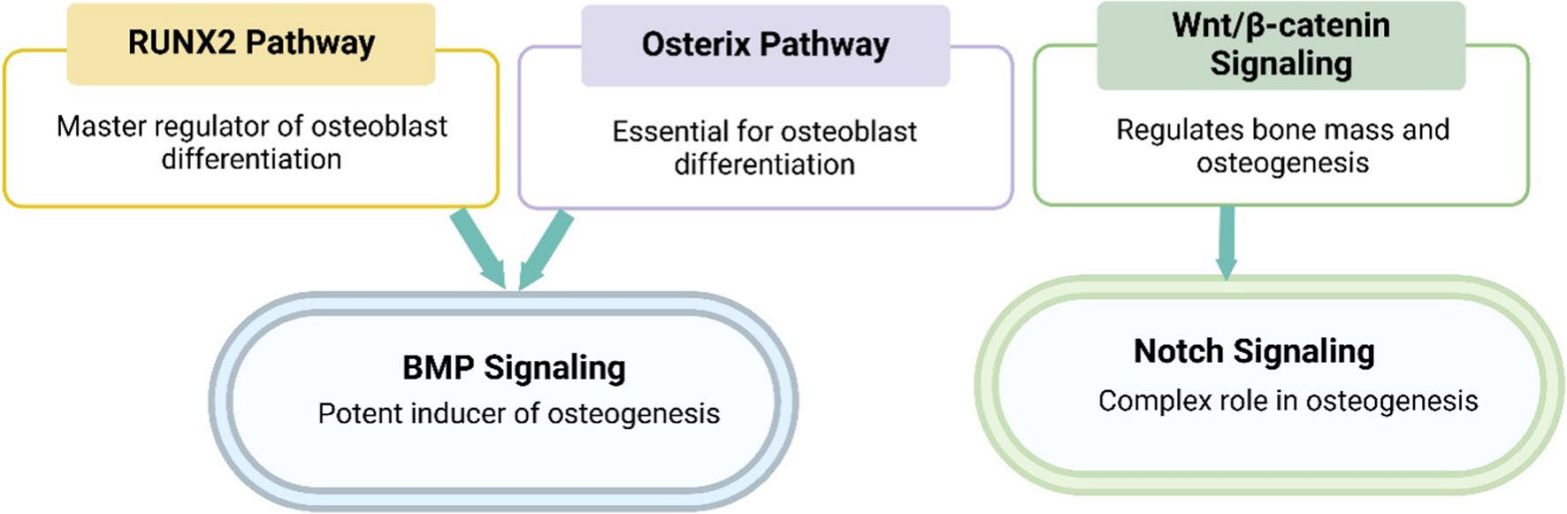
Key Molecular Pathways in Osteogenesis

Several molecular pathways play crucial roles in regulating osteogenesis (Fig. 2):

*RUNX2 pathway* RUNX2 (Runt-related transcription factor 2) is considered the master regulator of osteoblast differentiation. It acts as a transcription factor that controls the expression of key osteogenic genes, including osteocalcin, osteopontin, and type I collagen [30]. RUNX2 is regulated by various factors, including BMP (Bone Morphogenetic Protein) signaling, which enhances RUNX2 expression and activity; mechanical stress, which can activate RUNX2 through mechanotransduction pathways; and post-translational modifications, such as phosphorylation and acetylation, which modulate RUNX2 activity [31, 32].

*Osterix (OSX) pathway* Osterix, also known as Sp7, is a zinc finger-containing transcription factor that acts downstream of RUNX2 and is essential for osteoblast differentiation and bone formation. Osterix regulates the expression of numerous osteoblast-specific genes and is crucial for the commitment of preosteoblasts to mature osteoblasts [33]. Key aspects of the Osterix pathway include regulation by BMP and Wnt signaling pathways, interaction with other transcription factors, such as NFATc1, to regulate gene expression, and modulation by microRNAs, which can fine-tune Osterix expression and activity [34].

*Wnt/β-catenin signaling* The Wnt/β-catenin pathway is a critical regulator of bone mass and plays multiple roles in osteogenesis [35]. This pathway promotes osteoblast differentiation and proliferation, inhibits osteoclast formation and activity, and regulates bone mineralization and adaptation to mechanical loading [36].

*BMP signaling* Bone Morphogenetic Proteins (BMPs) are potent inducers of osteogenesis. The BMP pathway activates SMAD-dependent and SMAD-independent signaling cascades, induces the expression of RUNX2 and Osterix, and promotes the differentiation of mesenchymal stem cells into osteoblasts [37, 38].

*Notch signaling* The Notch pathway plays a complex role in osteogenesis, with stimulatory and inhibitory effects depending on the cellular context [39]. It regulates osteoblast differentiation and function, the bone marrow niche's maintenance, and the mesenchymal progenitor cells' regulation [40].

### Cellular mechanisms of osseointegration

Osseointegration involves a complex interplay of cellular mechanisms and processes by which bone tissue forms a direct structural and functional connection with an implant surface without intervening soft tissue. The process begins with an initial healing response, characterized by the forming of a blood clot and inflammatory response at the implant site, followed by recruiting mesenchymal stem cells and immune cells to the implant surface [41]. Osteoblast adhesion and differentiation occur, with osteoblast precursors attaching to the implant surface through integrin-mediated adhesion and differentiating into mature osteoblasts, influenced by surface topography and chemistry [42]. This is followed by osteoid deposition and mineralization, where osteoblasts secrete an unmineralized bone matrix [34] that gradually mineralizes to form mature bone tissue. Continuous bone remodeling around the implant adapts to mechanical stresses, maintaining a balance between bone formation by osteoblasts and resorption by osteoclasts [26]. Angiogenesis plays a crucial role in forming new blood vessels supporting bone growth and metabolism, facilitated by angiogenic factors secreted by osteoblasts and other cells at the implant site [43]. Implant surface interactions involve the adsorption of proteins from blood and extracellular fluid onto the implant surface, forming a bioactive layer that mediates cell attachment and differentiation. Finally, mechanotransduction processes convert mechanical stimuli into biochemical signals at the bone-implant interface, adapting the bone structure to distribute mechanical loads effectively [44]. Understanding these intricate mechanisms of osseointegration is essential for developing strategies to enhance bone regeneration and implant integration, offering multiple targets for therapeutic interventions, including photo-nanoparticles as drug delivery systems to modulate these processes.

## Phytonanoparticles as drug carriers for bone regeneration

### Advantages of phytonanoparticles in drug delivery

Phytonanoparticles offer a range of unique advantages as drug carriers for bone regeneration, setting them apart from conventional synthetic nanoparticles. Their plant-derived nature confers enhanced biocompatibility, significantly reducing the risk of adverse immune responses. Many phytonanoparticles are biodegradable, allowing for gradual breakdown and elimination from the body, which is crucial for ensuring long-term safety [45]. The plant extracts used in their synthesis often contain natural bioactive compounds that can synergistically enhance bone regeneration, adding an extra layer of therapeutic potential. This multifunctionality allows phytonanoparticles to serve simultaneously as drug carriers and bioactive agents, potentially amplifying the overall therapeutic effect [46, 47]. The natural capping agents present in these nanoparticles provide improved colloidal stability and prevent aggregation, enhancing their efficacy and shelf-life. From a production perspective, the green synthesis approach for phytonanoparticles is often more economical than traditional chemical synthesis methods, making them an attractive option for large-scale manufacturing. Furthermore, many phyto-nanoparticle synthesis methods have the potential for scalability, which is crucial for clinical translation and widespread application [48]. These combined advantages position phytonanoparticles as promising candidates for advancing bone regeneration therapies, offering a unique blend of biocompatibility, functionality, and production feasibility.

### Types of bioactive molecules delivered

*Growth factors* Growth factors play a crucial role in bone regeneration, and phytonanoparticles offer an effective delivery system for these proteins. Bone Morphogenetic Proteins (BMPs), particularly BMP-2 and BMP-7, are potent inducers of osteogenesis. Phytonanoparticles can protect these sensitive proteins from degradation and provide sustained release. Gold nanoparticles synthesized using *Cissus quadrangularis* extract have been shown to effectively deliver BMP-2, enhancing its osteogenic effects [49, 50]. Vascular Endothelial Growth Factor (VEGF) promotes angiogenesis, which is crucial for bone healing. Phytonanoparticles can co-deliver VEGF with other factors, creating a synergistic effect. Silver nanoparticles from *Ginkgo biloba* extract have demonstrated the ability to stimulate VEGF production and enhance vascularization in bone defects [51]. Fibroblast Growth Factor (FGF) stimulates angiogenesis and osteoblast proliferation. Zinc oxide nanoparticles synthesized using *Aloe vera* extract have been reported to deliver FGF-2 effectively, promoting bone formation and vascularization.

*Small molecules* Phytonanoparticles are excellent carriers for small-molecule drugs that can enhance bone regeneration. Dexamethasone, a synthetic glucocorticoid, promotes osteogenic differentiation. Gold nanoparticles synthesized using Epigallocatechin gallate from green tea have shown enhanced delivery of dexamethasone to osteoprogenitor cells, improving their differentiation [52]. Simvastatin, originally a cholesterol-lowering drug, has been shown to enhance bone formation. Phytonanoparticles derived from *Ocimum sanctum* have demonstrated improved delivery of simvastatin to bone tissue, enhancing its osteogenic effects [53]. Resveratrol, a natural polyphenol, has osteogenic and anti-inflammatory properties. Silver nanoparticles synthesized using grape seed extract have shown enhanced delivery of resveratrol, improving its bioavailability and osteogenic effects.

*Genes* Phytonanoparticles can serve as non-viral vectors for gene delivery in bone regeneration. RUNX2, the master regulator of osteoblast differentiation, can significantly enhance bone formation when delivered as a gene. Gold nanoparticles synthesized using *Ganoderma lucidum* extract have efficiently delivered RUNX2 plasmids to mesenchymal stem cells, improving their osteogenic differentiation. Osterix, essential for osteoblast differentiation and bone formation, can promote bone regeneration when its gene is delivered [54]. Chitosan nanoparticles modified with phytochemicals from *Gymnema sylvestre* have shown promising results in delivering Osterix-encoding plasmids to bone marrow stromal cells. Delivery of BMP genes, such as BMP-2 or BMP-7, can lead to sustained growth factor production. Copper nanoparticles synthesized using *Azadirachta indica* extract have efficiently delivered BMP-2 plasmids, resulting in prolonged BMP-2 expression in bone defect sites.

*Antibiotics* Phytonanoparticles can enhance the delivery and efficacy of antibiotics in bone regeneration. Vancomycin and Gentamicin are crucial for preventing or treating bone infections associated with implants or trauma. Silver nanoparticles produced from *Curcuma longa* extract have synergistic antibacterial effects when combined with vancomycin, enhancing its efficacy against biofilm-forming bacteria in bone infections [55]. Controlling inflammation is also crucial in bone healing, and phytonanoparticles can effectively deliver anti-inflammatory compounds. Curcumin, a natural anti-inflammatory compound with potential osteogenic effects, can be delivered more effectively using gold nanoparticles synthesized from *Curcuma longa* extract, improving its anti-inflammatory and osteogenic effects in bone tissue. Ibuprofen, used to manage inflammation during bone healing, can be optimized using phytonanoparticles [56]. Zinc oxide nanoparticles produced from *Ocimum basilicum* extract have shown controlled release of ibuprofen, providing sustained anti-inflammatory effects in bone regeneration models [57]. In conclusion, Phytonanoparticles offer versatile and practical delivery systems for various bioactive molecules crucial for bone regeneration, enhancing their stability, bioavailability, and targeted delivery in advanced bone tissue engineering applications.

### Mechanisms of drug loading and release

Phytonanoparticles offer diverse drug loading and release mechanisms, enhancing their potential in bone regeneration therapies. Drug loading methods include adsorption, where molecules attach to the nanoparticle surface, leveraging the large surface area and diverse functional groups from plant metabolites. Encapsulation incorporates drugs within the nanoparticle matrix during synthesis, benefiting from natural biomolecules that act as stabilizers [58]. Covalent bonding chemically attaches drugs to functional groups on the nanoparticle surface, utilizing the diverse phytochemicals present. Release mechanisms vary, including diffusion, where drugs gradually release from the nanoparticle matrix or surface; erosion, where release occurs as the nanoparticle degrades; and stimuli-responsive release, triggered by changes in pH, temperature, or enzymatic activity [59].

Controlled release strategies further enhance the versatility of phytonanoparticles in drug delivery. Layer-by-layer assembly creates multiple polyelectrolyte layers for sustained release, with phytonanoparticles serving as templates. Core–shell structures feature a drug-loaded core with a protective shell, often incorporating phytochemicals in the shell formation [60]. Porous structures allow drugs to be loaded into pores for gradual release, with some phytonanoparticles naturally forming such structures. These approaches offer great flexibility in designing drug-delivery systems tailored to specific therapeutic needs in bone regeneration [61]. The unique properties of phytonanoparticles, including their natural biomolecule coatings and potential for biodegradability, can be leveraged to optimize drug delivery kinetics and enhance therapeutic efficacy. Examples include curcumin adsorbed onto gold nanoparticles synthesized from ginger extract, doxorubicin encapsulated in neem-derived silver nanoparticles, and cisplatin covalently bound to green tea-derived gold nanoparticles, all demonstrating improved delivery and efficacy in various applications [62].

### Targeting strategies for bone tissue

**Fig. 3:**
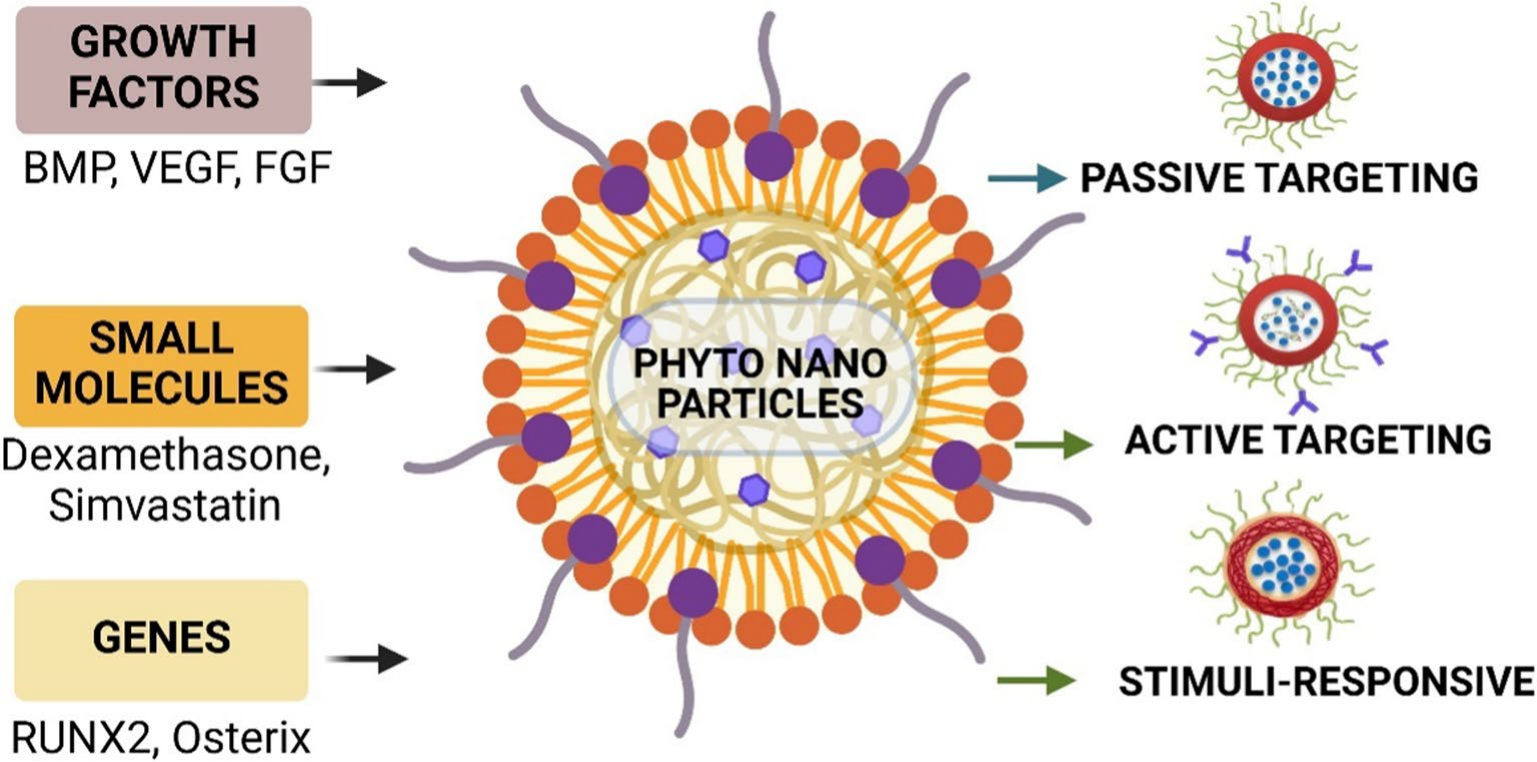
The figure illustrates the key components of phyto-nanoparticle drug delivery for bone regeneration, including types of bioactive molecules delivered and targeting strategies

Phytonanoparticles offer diverse targeting strategies for bone regeneration, leveraging both passive and active mechanisms. Passive targeting utilizes the Enhanced Permeability and Retention (EPR) effect and size-dependent properties, allowing nanoparticles to accumulate in areas of increased vascular permeability or penetrate bone tissue effectively. For instance, gold nanoparticles synthesized from turmeric extract have shown enhanced accumulation in osteoporotic bone tissue [63]. Active targeting involves surface modifications to increase bone affinity, such as bisphosphonate conjugation, peptide-mediated targeting, and aptamer-based approaches. Examples include green tea-derived gold nanoparticles conjugated with alendronate for improved bone targeting and holy basil-derived silver nanoparticles functionalized with bone-homing peptides for enhanced delivery to bone-forming surfaces [64, 65]. Advanced targeting strategies include cell-mediated approaches, stimuli-responsive targeting, and biomimetic methods (Fig. 3). Cell-mediated targeting uses mesenchymal stem cells or osteoblasts as carriers, while stimuli-responsive targeting designs nanoparticles to respond to specific bone microenvironment conditions, such as pH or enzyme activity. Biomimetic approaches involve mimicking natural bone components, like hydroxyapatite coatings or ECM protein functionalization, to improve targeting and integration [66, 67]. These diverse strategies, combined with the unique properties of phytonanoparticles, enable the development of precise and compelling drug delivery systems for bone tissue engineering. However, further research is needed to optimize these systems for clinical translation, enhancing drug loading efficiency, fine-tuning release kinetics, and improving targeting specificity to bone tissue.

## Enhancing osteogenesis and osseointegration with phytonanoparticles

### Gold nanoparticles: synthesis, characterization, and osteogenic effects

Gold nanoparticles (AuNPs) synthesized using plant extracts have demonstrated significant potential in enhancing osteogenesis and osseointegration. The synthesis of these nanoparticles typically involves reducing chloroauric acid (HAuCl4) with plant extracts, as exemplified by Nune et al. [68], who used Cinnamomum zeylanicum (cinnamon) extract to produce spherical AuNPs with an average size of 25 nm. Characterization of these nanoparticles often involves Transmission Electron Microscopy (TEM) for size and morphology confirmation and UV–visible spectroscopy to verify AuNP formation through the characteristic surface plasmon resonance peak. The osteogenic effects of plant-derived AuNPs have been well-documented in various studies. Guan et al., [69] showed that AuNPs synthesized from *Eucalyptus globulus* leaf extract significantly enhanced the proliferation of MC3T3-E1 osteoblast-like cells. Improved calcium deposition and alkaline phosphatase (ALP) activity were reported in human mesenchymal stem cells treated with AuNPs produced from Panax ginseng root extract [70]. Rich et al. [71] observed the upregulation of key osteogenic genes (RUNX2, Osterix, and Osteocalcin) in human adipose-derived stem cells exposed to AuNPs. In vivo studies have also yielded promising results, demonstrating significantly higher bone-to-implant contact and new bone formation in a rat model using titanium implants coated with AuNPs [72].

### Silver nanoparticles: antimicrobial properties and bone regeneration

Silver nanoparticles (AgNPs) have garnered significant attention in bone regeneration applications due to their well-established antimicrobial properties and emerging osteogenic potential. The synthesis of AgNPs from *Angelica sinensis* extract has demonstrated antimicrobial properties [73]. Characterization of these nanoparticles typically involves Transmission Electron Microscopy (TEM), Dynamic Light Scattering (DLS) for size confirmation, and X-ray Diffraction (XRD) to verify their crystalline nature. The antimicrobial properties of plant-derived AgNPs have been extensively studied, demonstrating broad-spectrum activity against common bone infection pathogens using AgNPs synthesized from *Azadirachta indica* leaf extract [74]. Ni et al.[75] showed that AgNPs produced from *Ginkgo biloba* leaf extract inhibited biofilm formation. Beyond their antimicrobial effects, AgNPs have shown promising bone regeneration properties. AgNPs synthesized using *Panax notoginseng* root extract promoted osteogenic differentiation of bone marrow mesenchymal stem cells by activating the BMP/Smad signaling pathway [76]. The angiogenic potential of AgNPs produced from *Salvia miltiorrhiza* extract enhanced vascular endothelial growth factor (VEGF) expression in osteoblasts and promoted angiogenesis [77].

### Other metal and metal oxide nanoparticles

Several other metal and metal oxide nanoparticles have shown potential in enhancing osteogenesis and osseointegration:

### Zinc oxide nanoparticles (ZnO NPs)

Zinc oxide nanoparticles have emerged as promising candidates for bone regeneration applications [78]. Abel et al. [79] demonstrated a synthesis method using *Moringa oleifera* leaf extract to produce ZnO NPs. These plant-derived ZnO NPs have shown significant osteogenic potential. In a study by Senthilkumar & Thirumal [80], ZnO NPs synthesized using *Camellia sinensis* extract exhibited a remarkable ability to enhance alkaline phosphatase (ALP) activity and mineralization. This suggests that ZnO NPs can effectively promote the differentiation of osteoblasts and the formation of new bone matrix, making them valuable tools in bone tissue engineering.

### Copper nanoparticles (Cu NPs)

Copper nanoparticles have attracted attention for their potential in promoting angiogenesis, a crucial process in bone regeneration. Ben Mosbah et al. [81] reported a green synthesis method for Cu NPs using *Punica granatum* (pomegranate) peel extract, highlighting the versatility of plant-based nanoparticle synthesis. The angiogenic effects of these Cu NPs were demonstrated in a study by Li et al. (2023). Cu NPs produced using *Salvia miltiorrhiza* extract significantly promoted angiogenesis in a rat femoral defect model. This effect was attributed to the upregulation of key angiogenic factors HIF-1α and VEGF, suggesting that Cu NPs could enhance blood vessel formation during bone healing.

### Iron oxide nanoparticles (Fe3O4 NPs)

Iron oxide nanoparticles offer unique advantages in bone regeneration due to their magnetic properties, demonstrating another example of plant-based nanoparticle production [82]. When combined with magnetic field exposure, these nanoparticles' potential in bone regeneration significantly enhanced the osteogenic differentiation of bone marrow mesenchymal stem cells (BMSCs) [83, 84]. This magnetic field-assisted osteogenesis opens up new possibilities for controlled and targeted bone regeneration therapies, potentially allowing for non-invasive stimulation of bone formation in specific areas. The diverse potential of metal and metal oxide nanoparticles in bone regeneration, from promoting osteoblast differentiation and mineralization to enhancing angiogenesis and enabling magnetic field-assisted therapies [85].

### Comparative analysis of different phytonanoparticles for bone applications

To provide a comprehensive comparison of different phytonanoparticles for bone applications, we can analyze their performance across several key parameters (Table 2):

**Table 2:** Comparison of phytonanoparticles for bone applications, highlighting their relative strengths and limitations across key properties relevant to bone regeneration

Comparison of phyto-nanoparticles					
Properties	Gold NPs	Silver NPs	Zinc oxide NPs	Copper NPs	Iron oxide NPs
Osteogenic potential	High	Moderate	Good	Moderate	Moderate
Antimicrobial activity	Low	High	Moderate	Moderate	Low
Angiogenic effects	Moderate	Good	Moderate	High	Low
Biocompatibility	High	Moderate	Good	Moderate	Good
Multifunctionality	Moderate	High	Moderate	High	High (magnetic)
Ease of synthesis	High	High	Moderate	Moderate	Moderate
Key advantage	Strong osteogenic effects	Antimicrobial + osteogenic	Balanced properties	Strong angiogenic effects	Magnetic field-assisted osteogenesis
Main limitation	Limited antimicrobial activity	Potential cytotoxicity	Moderate overall effects	Potential cytotoxicity	Limited intrinsic bioactivity
Best suited for	Enhancing bone formation	Infection prevention + bone healing	General bone regeneration	Vascularization in bone defects	Controlled, externally triggered bone regeneration

#### Osteogenic and angiogenic potential

Gold nanoparticles (AuNPs) demonstrate the highest osteogenic potential among phytonanoparticles, strongly affecting osteoblast proliferation and differentiation [68, 86]. This makes them particularly suitable for applications requiring robust bone formation. Silver nanoparticles (AgNPs), while showing moderate osteogenic effects, excel in promoting angiogenesis, a crucial factor in bone healing [87]. Zinc oxide nanoparticles (ZnO NPs) exhibit good osteogenic potential, particularly in enhancing alkaline phosphatase (ALP) activity and mineralization [88]. Copper nanoparticles (Cu NPs) stand out for their potent angiogenic effects, which can be particularly beneficial in large bone defects where vascularization is challenging [89]. The combination of these properties suggests that a multi-nanoparticle approach might be optimal for comprehensive bone regeneration strategies.

#### Antimicrobial properties and biocompatibility

AgNPs demonstrate the strongest and broadest antimicrobial activity, making them invaluable for preventing infection in bone healing applications [90]. ZnO NPs also show significant antimicrobial effects, though generally less potent than AgNPs [91]. AuNPs have limited intrinsic antimicrobial activity and can be functionalized to enhance this property [92]. Regarding biocompatibility, AuNPs generally show the highest tolerability, while ZnO NPs and iron oxide nanoparticles (Fe3O4 NPs) demonstrate good biocompatibility at lower concentrations [93]. AgNPs and Cu NPs may exhibit dose-dependent cytotoxicity and require careful dosing. This balance between antimicrobial efficacy and biocompatibility is crucial in designing safe and effective bone regeneration therapies [94].

#### Multifunctionality and Synthesis Considerations

AgNPs offer a unique combination of antimicrobial, osteogenic, and angiogenic properties, making them versatile candidates for bone regeneration applications [95]. Cu NPs provide both osteogenic and angiogenic solid effects, while Fe3O4 NPs offer the additional benefit of magnetic field-assisted osteogenesis. This multifunctionality can be advantageous in addressing multiple aspects of bone healing simultaneously. Regarding synthesis and characterization, AuNPs and AgNPs are generally the easiest to produce and analyze due to their distinctive optical properties [96].

The choice of photo-nanoparticle for bone regeneration applications should be based on the specific requirements of each case, considering factors such as osteogenic potential, antimicrobial needs, angiogenic effects, and biocompatibility. Combination strategies, leveraging the strengths of different nanoparticles, may provide synergistic benefits in bone tissue engineering applications. Future research should focus on optimizing these combinations and addressing any potential limitations to harness the potential of phytonanoparticles in bone regeneration fully.

**Comparison of different phytonanoparticles for bone applications**

## Advanced strategies in phytonanoparticle design for bone regeneration

**Fig. 4:**
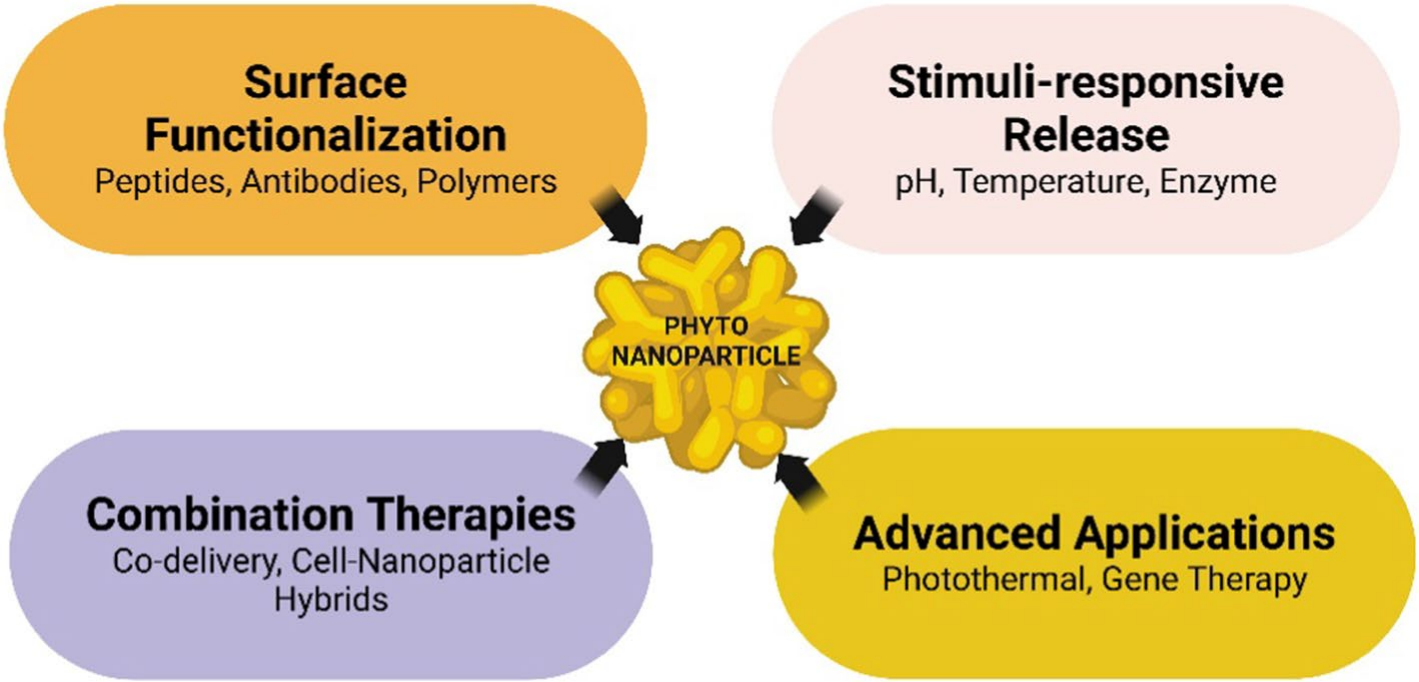
Illustrating the key advanced strategies in phyto-nanoparticle design for bone regeneration, showing how various techniques and approaches converge to enhance the functionality and efficacy of phytonanoparticles

The photo-nanoparticle design for bone regeneration is rapidly evolving, with researchers developing increasingly sophisticated strategies to enhance their efficacy and functionality (Fig. 4).

### Surface functionalization techniques

Surface functionalization of phytonanoparticles enhances their performance in bone regeneration by improving targeting, cellular uptake, and bioactivity. Four main techniques are employed: biomolecule conjugation, polymer coating, biomimetic mineralization, and dual functionalization. Biomolecule conjugation involves attaching specific molecules like bone-targeting peptides or antibodies to improve targeting and biological activity [97]. Polymer coating enhances stability and circulation time using polymers like PEG or chitosan. Biomimetic mineralization coats nanoparticles with bone-like minerals to improve integration with bone tissue. Dual functionalization combines multiple techniques for multi-functional nanoparticles. These approaches create specialized nanoparticles tailored for specific aspects of bone regeneration, from targeted delivery to enhanced tissue integration [98]. Future research will likely focus on developing more sophisticated strategies further to improve efficacy and specificity in bone regeneration applications.

### Stimuli-responsive release mechanisms

Stimuli-responsive release mechanisms incorporated into phyto-nanoparticle designs offer precise control over the release of therapeutic agents in response to specific environmental cues. This advanced approach enhances the efficacy and specificity of drug delivery in bone regeneration applications. Stimuli-responsive release mechanisms in phyto-nanoparticle designs offer precise control over therapeutic agent release in bone regeneration applications [99]. These systems respond to specific environmental cues, enhancing drug delivery efficacy and specificity. Five main types are identified: pH-responsive systems, exploiting pH variations in physiological environments; temperature-sensitive release, responding to local temperature changes; enzyme-activated systems, triggered by specific tissue enzymes; magnetic field-triggered release, allowing non-invasive external control; and redox-responsive systems, utilizing differences in redox potentials. Each mechanism offers unique advantages: pH-responsive systems target active bone resorption areas, temperature-sensitive systems respond to inflammation, enzyme-activated systems focus on active remodeling sites, magnetic field-triggered systems provide external control, and redox-responsive systems enable intracellular delivery [100, 101]. By incorporating these elements, researchers create sophisticated drug delivery systems responding dynamically to the complex environment of healing bone tissue, potentially enhancing the efficacy and precision of bone regeneration therapies and opening new avenues for personalized treatments.

### Combination therapies

Combination therapies utilizing phytonanoparticles offer synergistic effects that enhance bone regeneration outcomes. Fundamental approaches include drug co-delivery systems, cell-nanoparticle hybrids, photothermal therapy combined with drug delivery, gene and drug co-delivery, and antimicrobial and osteogenic dual functionality [102, 103]. These strategies simultaneously address multiple aspects of bone healing, such as promoting osteogenesis, angiogenesis, and infection prevention. Drug co-delivery systems use phytonanoparticles to carry multiple agents, enhancing osteogenic differentiation and bone formation. Cell-nanoparticle hybrids combine stem cells with therapeutic nanoparticles, leveraging cellular targeting abilities while enhancing regenerative capabilities [104]. Photothermal therapy with drug delivery combines physical and chemical treatments for improved bone regeneration. Gene and drug co-delivery allow fine-tuning of cellular responses, while dual-functional approaches simultaneously address infection prevention and bone regeneration [105]. These advanced combinations represent significant progress in phyto-nanoparticle design, offering more sophisticated and personalized treatment strategies for complex bone healing challenges.

## Preclinical studies

The development of phytonanoparticles for bone regeneration has progressed through various stages of preclinical research, with some approaches beginning to enter early clinical evaluation.

Numerous in vitro studies have demonstrated the potential of phytonanoparticles to enhance osteoblast proliferation and differentiation. A study investigating gold nanoparticles (AuNPs) synthesized using *Coptis chinensis* extract showed enhanced proliferation and ALP activity in MC3T3-E1 cells, with optimal effects at 10 μg/mL[106]. However, this study did not explore long-term effects or potential cytotoxicity at higher concentrations. Another study examining silver nanoparticles (AgNPs) produced from *Azadirachta indica* extract found increased osteogenic differentiation of human mesenchymal stem cells (hMSCs), evidenced by the upregulation of RUNX2 and Osteocalcin[107]. While this study provided comprehensive gene expression data, it lacked functional assays to confirm enhanced mineralization. A third study evaluating zinc oxide nanoparticles (ZnO NPs) synthesized using *Camellia sinensis* extract demonstrated a dose-dependent increase in osteoblast proliferation and ALP activity, with optimal effects at 50 μg/mL[108].

In vivo studies have provided valuable insights into the efficacy of phytonanoparticles in more complex biological environments. A study testing gold nanoparticles (AuNPs) synthesized from *Panax ginseng* extract in a rat calvarial defect model showed significantly enhanced bone formation and defect closure after eight weeks compared to untreated controls [109]. Another study investigating silver nanoparticles produced using *Ginkgo biloba* extract in a rat femoral fracture model found accelerated fracture healing [110]. A study evaluating titanium implants coated with zinc oxide nanoparticles (ZnO NPs) synthesized from *Aloe vera* extract in an animal model demonstrated improved bone-to-implant contact [111].

## Challenges and future perspectives

While phytonanoparticles are promising for bone regeneration applications, several challenges must be addressed to realize their full potential in clinical settings. The transition from laboratory-scale synthesis to industrial production presents issues such as batch-to-batch variability due to inconsistencies in plant extract composition, which can be addressed by developing standardized cultivation and extraction protocols. Maintaining nanoparticle size distribution and morphology during large-scale production is another challenge that may be overcome by investigating continuous flow synthesis methods and microfluidic approaches [24, 112]. Efficient separation of nanoparticles from unreacted precursors and plant debris in large-scale production poses difficulties. Still, it can be improved by developing advanced purification techniques such as tangential flow filtration and implementing in-line characterization methods. Maintaining the long-term stability of phytonanoparticles without aggregation or loss of bioactivity is also a concern, which may be resolved by investigating optimal storage conditions and developing stabilizing formulations to enhance shelf-life [17, 113].

The unique nature of phytonanoparticles poses several regulatory, safety, economic, and environmental challenges. Determining the appropriate regulatory pathway for these particles, which have botanical products and nanomaterial characteristics, requires engagement with regulatory agencies to develop specific guidance. Evaluating long-term safety and potential toxicity, considering unique properties and bioaccumulation potential, necessitates comprehensive toxicological studies. Standardization of characterization methods and understanding long-term biodistribution and clearance are also crucial. Economically, ensuring cost-effective production compared to traditional methods requires process optimization and comprehensive cost–benefit analyses [114, 115]. Environmental considerations include assessing and minimizing the production footprint, implementing green chemistry principles, ensuring sustainable sourcing of plant materials, and managing waste effectively.

Future research directions for phytonanoparticles in bone regeneration include exploring advanced synthesis techniques, investigating combination therapies, developing smart delivery systems, pursuing personalized medicine approaches, creating non-invasive monitoring methods, and designing biomimetic nanocomposites. These advancements aim to enhance control over nanoparticle properties, improve efficacy in treating complex bone defects, enable targeted and controlled release of therapeutic agents, optimize treatment outcomes based on patient-specific needs, improve treatment efficacy assessment, and enhance bone tissue integration in complex defects. Potential clinical applications span various fields, including dental and maxillofacial surgery, orthopedic trauma, spinal fusion, osteoporosis management, and bone cancer therapy [116]. These applications range from enhancing osseointegration in dental implants to targeted delivery of osteoanabolic agents for osteoporosis and combination therapies for bone cancer. While phytonanoparticles are promising, significant scalability, regulation, and clinical translation challenges must be addressed through focused research efforts and collaboration between academia, industry, and regulatory bodies. The future of photo-nanoparticles in bone regeneration lies in developing sophisticated, tailored, and multifunctional systems that meet the complex needs of bone tissue engineering and regenerative medicine.

## Conclusion

Phytonanoparticles represent a promising frontier in bone regeneration, offering unique biocompatibility, multifunctionality, and sustainability advantages. This review has explored their synthesis, characterization, mechanisms of action, and applications in enhancing osteogenesis and osseointegration. Preclinical studies have demonstrated their potential to accelerate bone formation, improve fracture healing, and enhance implant integration. While early clinical studies show promise, significant challenges remain in standardization, scale-up, and regulatory approval. Future research should focus on developing advanced formulations, exploring personalized approaches, and conducting long-term safety and efficacy studies. Integrating phytonanoparticles with other regenerative strategies may lead to more comprehensive bone regeneration solutions. Interdisciplinary collaboration will be crucial as the field progresses in translating these nature-inspired nanomaterials into practical clinical applications. With continued research and development, phytonanoparticles have the potential to revolutionize bone regeneration therapies, offering innovative solutions for patients with bone injuries and disorders.

## Data Availability

No datasets were generated or analysed during the current study.
